# Cross-Axis Coupling Effects in Single-Axis Nuclear Magnetic Resonance Gyroscopes

**DOI:** 10.3390/s20030734

**Published:** 2020-01-29

**Authors:** Zhiguo Wang, Yi Zhang, Xiang Zhan, Qiyuan Jiang, Hui Luo

**Affiliations:** College of Advanced Interdisciplinary Studies, and Interdisciplinary Center of Quantum Information, National University of Defense Technology, Changsha 410073, China; abnormal_zy@163.com (Y.Z.); zhanxiang09@163.com (X.Z.); qiyuanjiang1990@163.com (Q.J.); luohui.luo@163.com (H.L.)

**Keywords:** nuclear magnetic resonance gyroscope, cross-axis coupling, magnetic field, rotation rate, frequency shift

## Abstract

Nuclear magnetic resonance gyroscopes (NMRGs) may be operated in an environment with violent vibration that usually contains both linear components and angular components. To analyze the influence of angular vibration on an NMRG, cross-axis coupling effects are studied. The cross-axis rotation rates induce an equivalent magnetic field. Its influence can be described by the Bloch equations. The approximate frequency shift and amplitude of the spin oscillator with an equivalent magnetic field in the cross-axis were obtained, which was validated by numerical simulation. The findings show that the angular vibration component leads to a remarkable error for the NMRG. When the angular vibration frequency is near the Larmor frequency, the oscillation frequency of the spins may be locked to the angular vibration frequency, destroying the NMRG’s ability to measure rotation rates. The cross-axis coupling problem should be considered in the design of an NMRG and corresponding inertial navigation systems.

## 1. Introduction

A nuclear magnetic resonance gyroscope (NMRG) measures rotation rates through the detection of the Larmor precession frequency of atomic spins in a static magnetic field [1,2,3,4]. Its physical foundation can be traced back to the pioneering work done by Larmor in 1895, Rabi in 1938, Bloch et al. in 1946 [5,6]. Leete filed a patent application on NMRG in 1952, which was assigned to the General Electric Company. Since then, several other companies conducted research on the NMRG. Both Singer and Litton had demonstrated NMRG prototypes with navigation grade in the 1980s [7]. However, compared with optical gyroscopes developed in the same period, the NMRG did not show enough advantages. Therefore, research on the NMRG entered a low tide. 

The turnaround took place in the 2000s when micro-fabrication technology became more and more mature [7]. NMRGs have attracted considerable attention due to potential advantages including small size, weight and power. It is believed that the NMRG is an ideal sensor for inertial applications, since it has no mechanical part and is thus insensitive to mechanical shock and vibration [6,7,8]. The NMRG has already achieved near navigation-grade performance with a volume of 10 cm^3^ [5], indicating a bright prospect for mass applications.

The NMRG has the greatest sensitivity to the axis along the applied static field, but it can be affected by cross-axis rotation rates as well [9,10]. That is, the NMRG is not a true single-axis gyroscope. Physically, the NMRG is essentially a spin oscillator that is easily affected by a magnetic field, regardless of it being a true or equivalent magnetic field [9,10,11,12]. The magnetic field shield suppresses the external magnetic field but cannot suppress the equivalent magnetic field due to mechanical rotation. The cross-axis coupling effects for a cryogenic ^3^He gyroscope have been investigated in detail [9,10]. The relaxation time for ^3^He can be as long as 140 h, which makes the relaxation in the dynamical equation of spins negligible, and the ^3^He gyroscope can be operated in an open-loop mode [10]. It was clear that cross-axis rotation will degrade the performance of an NMRG. Recently, it was found that an NMRG using ^129^Xe and ^131^Xe is easily miniaturized, which has attracted considerable attention [2]. However, the relaxation time of ^129^Xe and ^131^Xe is much shorter than that of cryogenic ^3^He. As a result, it is better to operate such an NMRG in a closed-loop mode, where a feedback driving field is applied [11]. Due to these differences, the previous analysis cannot be directly applied to miniature NMRGs. Miniature NMRGs have been mainly developed for use in strap-down inertial navigation systems, which not only need high precision but also a low cross-axis sensitivity [13]. Therefore, it is important to analyze the cross-axis coupling effects in an NMRG based on ^129^Xe and ^131^Xe.

In this paper, we established a theoretical model for the spin oscillator to analyze the cross-coupling effects in a ^129^Xe/^131^Xe NMRG. We find that these effects lead to a shift in the Larmor frequency, i.e., a measurement error in the NMRG. When the mechanical vibration contains a cross-axis rotation component near the Larmor frequency, the error will be very large. Moreover, this may result in the oscillation frequency being locked to the mechanical vibration rate, which destroys the NMRG’s ability to measure the rotation rate. These results are of significance to the design of an NMRG and its corresponding strap-down inertial navigation system.

## 2. Theoretical Analysis

### 2.1. Principle of an NMRG

**Figure 1:**
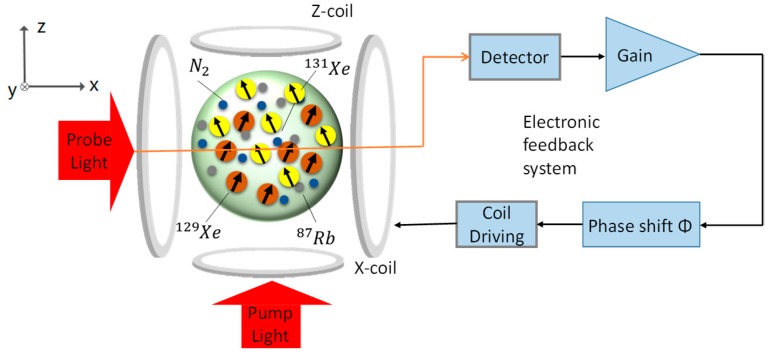
Principle of a dual-species spin oscillator.

An NMRG using ^129^Xe and ^131^Xe is, in fact, a dual-species spin oscillator, as is shown in Figure 1 [1,11,14,15]. A vapor cell contains a mixture of gases, including ^129^Xe/^131^Xe, N_2_ and so on. To obtain angular momentum from the pump light, an excess amount of ^87^Rb is filled into the vapor cell. When heated, the ^87^Rb will vaporize and absorb pump light. The pump light is circularly polarized, so each photon carries angular momentum equal to one ℏ in the propagating direction. After taking in the circularly polarized photons, the spin of the ^87^Rb atom will align along the z-direction, i.e., become polarized. Through spin-exchange collisions, the angular momentum transfers from the ^87^Rb spins to the ^129^Xe/^131^Xe nuclear spins. As a result, the ^129^Xe/^131^Xe nuclear spins become polarized and give rise to a magnetization M. A linearly polarized probe light transmits through the cell and interacts with the polarized ^87^Rb atoms. The polarization plane of the transmission light varies with the magnetic field sensed by the ^87^Rb atoms. Therefore, the magnetization of the nuclear spins can be detected. The output of the detector is sent to a signal processing system, which is used to obtain the magnetic field in the x and y directions. The *y*-axis magnetization component $M_{y}$ is amplified in the gain module, shifted by a phase of Φ, and then applied to drive the X-coil. For an appropriate gain and phase shift, the nuclear spins will precess continuously. More details for the NMRG can be found in [1].

The spin oscillator shown in Figure 1 actually has two spin oscillators: a ^129^Xe oscillator and a ^131^Xe oscillator. The ^129^Xe and ^131^Xe spins show negligible interaction through direct spin interaction, but coupling through the driving coil can occur. The driving fields of ^129^Xe and ^131^Xe influence each other, but this is not our concern here. In the following derivation, we will take the ^129^Xe spin oscillator as an example to describe the operating principle. 

The motion of the spins satisfies the following Bloch equation [10,12]:

(1) $$\frac{d}{dt}M=M\times \left(\gamma B+\Omega \right),$$

where M is the magnetization of the spins, γ is the gyromagnetic ratio, B is the magnetic field and Ω is the rotation rate of the gyroscope with respect to the inertial frame.

Equation (1) shows that the rotation has the same effect as a magnetic field $B_{eff}=\Omega /\gamma$. Thus, we will discuss cross-coupling effects using the following equation:

(2) $$\frac{d}{dt}M=M\times \gamma B.$$

Taking the relaxation time into account, Equation (2) can be expanded into the motion equation for the components of the magnetization:

(3) $$\left\{ \begin{array}{l} \frac{dM_{x}}{dt}=\gamma (M_{y}B_{z}-M_{z}B_{y})-\frac{M_{x}}{T_{2}}\\ \frac{dM_{y}}{dt}=\gamma (M_{z}B_{x}-M_{x}B_{z})-\frac{M_{y}}{T_{2}}\\ \frac{dM_{z}}{dt}=\gamma (M_{x}B_{y}-M_{y}B_{x})+\frac{M_{0}-M_{z}}{T_{1}} \end{array}\right.,$$

where $M_{x}$, $M_{y}$ and $M_{z}$ are the three components of the magnetization M, respectively; $B_{x}$, $B_{y}$ and $B_{z}$ are the three components of the magnetic field, respectively; $T_{1}$ and $T_{2}$ are the longitudinal spin relaxation time and the transverse relaxation time, respectively. 

Defining a complex vector $M_{+}=M_{x}+iM_{y}$, and, from Equation (3), we can obtain:

(4) $$\left\{ \begin{array}{l} \frac{dM_{+}}{dt}=-i\gamma B_{z}M_{+}+i\gamma M_{z}\left(B_{x}+iB_{y}\right)-\frac{M_{+}}{T_{2}}\\ \frac{dM_{z}}{dt}=\gamma (M_{x}B_{y}-M_{y}B_{x})+\frac{M_{0}-M_{z}}{T_{1}} \end{array}\right..$$

To achieve analytical expressions for the oscillation frequency and amplitude for the ^129^Xe spin oscillator, we posit the following hypothesis. A static magnetic field with magnitude $B_{z}=B_{0}$ is applied along the *z*-axis, and a field $B_{x}^{d}=2D\cos\left(\omega_{0}t\right)$ is applied along the *x*-axis to drive the spins. The feedback system has limited bandwidth so that the components with a frequency out of the range of $\omega \pm \Delta \omega_{f}$ are filtered out. Here, $\Delta \omega_{f}$ is the bandwidth of the filter. Through adjusting the phase shift and gain in the feedback loop, the spin magnetization precesses about the *z*-axis with frequency $\omega_{0}$ in a clockwise manner. Therefore, we can write the following expressions:

(5) $$\begin{array}{c} M_{x}=M_{x0}\cos\left(\omega_{0}t\right)+M_{y0}\sin\left(\omega_{0}t\right)\\ M_{y}=-M_{x0}\sin\left(\omega_{0}t\right)+M_{y0}\cos\left(\omega_{0}t\right) \end{array},$$

where $M_{x0}$ and $M_{y0}$ are the components in the *x* and *y*-axes at t=0, respectively.

With a rotatingwave approximation, we can describe the driving magnetic field for ^129^Xe as follows:

(6) $$\begin{array}{l} B_{x}^{d}=D\cos\left(-\omega_{0}t\right)\\ B_{y}^{d}=D\sin\left(-\omega_{0}t\right) \end{array},$$

Inserting Equations (5) and (6) into Equation (4), we have:

(7) $$\begin{array}{l} M_{+0}=i\gamma DM_{z0}T_{2}\\ \gamma DM_{y0}=\frac{M_{0}-M_{z0}}{T_{1}} \end{array},$$

where $M_{+0}=M_{x0}+iM_{y0}$ and $M_{z0}$ is the $M_{z}$ in the steady state.

### 2.2. Modeling of a Spin Oscillator with a Cross-Axis-Rotation-Equivalent Magnetic Field

The influence of the cross-axis-rotation can be modeled by a transverse magnetic field; we might as well call this the cross-axis-rotation-equivalent (CARE) magnetic field. Without loss of generality, the CARE magnetic field can be described as follows:

(8) $$\begin{array}{l} B_{x}^{e}=\sum_{n}B_{n}\cos\left(-\omega_{n}t-\varphi_{n}\right)\\ B_{y}^{e}=\sum_{n}B_{n}\sin\left(-\omega_{n}t-\varphi_{n}\right) \end{array},$$

where $B_{n}$, $\omega_{n}$ and $-\varphi_{n}$ are the amplitude, angular frequency and phase of the n-th component, respectively. 

With the CARE magnetic field, the motion of the spin oscillator still satisfies Equation (4), where $B_{x}=B_{x}^{d}+B_{x}^{e},\;B_{y}=B_{y}^{d}+B_{y}^{e}$. We assume that the spins still precess around the *z*-axis but that the angular frequency of $B_{x}^{d}$ and $B_{y}^{d}$ changes from $\omega_{0}$ to ω because of the CARE magnetic field; thus, we obtain the following expressions for the solutions of Equation (4):

(9) $$\begin{array}{l} M_{+}=\left(M_{⊥0}+M_{⊥}^{*}\right)e^{-i\omega t}=M_{⊥}e^{-i\omega t}\\ M_{z}=M_{z0}+M_{z}^{*} \end{array},$$

where $M_{⊥}^{*}$ and $M_{z}^{*}$ are variations of the transverse and longitudinal magnetization due to the CARE magnetic field.

Inserting Equations (8) and (9) into Equation (4), we have:

(10) $$\begin{array}{l} \frac{dM_{+}}{dt}+i\gamma B_{0}M_{+}+\frac{M_{+}}{T_{2}}=i\gamma M_{z}\left(De^{-i\omega t}+\sum_{n}B_{n}e^{-i\omega_{n}t-\varphi_{n}}\right)\\ \frac{dM_{z}}{dt}=-\gamma DM_{y0}+\gamma M_{x0}\sum_{n}B_{n}\sin\left(\omega t-\omega_{n}t-\varphi_{n}\right)-\gamma M_{y0}\sum_{n}B_{n}\cos\left(\omega t-\omega_{n}t-\varphi_{n}\right)+\frac{M_{0}-M_{z}}{T_{1}} \end{array}.$$

Substituting Equations (7) and (9) into Equation (10), we have:

(11) $$M_{z}^{*}\approx -\frac{1}{2}\gamma \sum_{n}B_{n}\left[\frac{M_{y0}+iM_{x0}}{1/T_{1}+i\left(\omega -\omega_{n}\right)}e^{i\left(\omega -\omega_{n}\right)t-i\varphi_{n}}-\frac{iM_{x0}-M_{y0}}{1/T_{1}-i\left(\omega -\omega_{n}\right)}e^{-i\left(\omega -\omega_{n}\right)t+i\varphi_{n}}\right],$$

and:

(12) $$\frac{dM_{⊥}}{dt}+\frac{M_{⊥}}{T_{2}}-i\left(\omega -\omega_{0}\right)M_{⊥}=i\gamma \left[M_{z0}+M_{z}^{*}\right]\left(D+\sum_{n}B_{n}e^{i\left(\omega -\omega_{n}\right)t-i\varphi_{n}}\right).$$

Since the response of $M_{⊥}$ to an alternating magnetic field with angular frequency larger than $1/T_{2}$ is small, we might as well neglect terms that contain $e^{i\left(\omega -\omega_{n}\right)t-i\varphi_{n}}$ in Equation (12). As a result, Equation (12) can be reduced to:

(13) $$\frac{d}{dt}M_{⊥}^{*}+\frac{1}{T_{2}}M_{⊥}^{*}-i\left(\omega -\omega_{0}\right)\left(M_{⊥0}+M_{⊥}^{*}\right)=-\left(M_{⊥0}+M_{⊥}^{*}\right)\sum_{n}\frac{1}{2}{\left(\gamma B_{n}\right)}^{2}\frac{1/T_{1}+i\left(\omega -\omega_{n}\right)}{{\left(1/T_{1}\right)}^{2}+{\left(\omega -\omega_{n}\right)}^{2}},$$

Using the approximation that $M_{⊥}^{*}$ varies very slowly with time, we obtain the following equation from Equation (13):

(14) $$\omega =\omega_{0}+\sum_{n}\frac{1}{2}{\left(\gamma B_{n}\right)}^{2}\frac{\left(\omega -\omega_{n}\right)}{{\left(1/T_{1}\right)}^{2}+{\left(\omega -\omega_{n}\right)}^{2}}\approx \omega_{0}+\sum_{n}\frac{1}{2}{\left(\gamma B_{n}\right)}^{2}\frac{\left(\omega_{0}-\omega_{n}\right)}{{\left(1/T_{1}\right)}^{2}+{\left(\omega_{0}-\omega_{n}\right)}^{2}}.$$

In a practical case, the CARE field is usually small, so we replace the oscillating frequency ω with $\omega_{0}$ in the last term of Equation (14) to avoid the difficulty in solving the oscillating frequency. The validity of the approximation can be proved in the following simulation.

Equation (14) gives the Bloch–Siegert shift formula for spin oscillators [16,17,18]. It is clear that when $\omega_{0}>\omega_{n}$, $\Delta \omega =\omega -\omega_{0}>0$ and vice versa. Thus, the rotating field $B_{n}$ pushes the oscillation frequency farther away from $\omega_{n}$. When $\omega_{n}=-\omega_{0}$ and $B_{n}=D$, Equation (14) can be written as:

(15) $$\omega =\omega_{0}+\frac{{\left(\gamma D\right)}^{2}}{4\omega_{0}}.$$

This is just the case of a Bloch–Siegert shift for only one linear driving magnetic field. When $\omega_{n}=0$ and $B_{n}=D$, Equation (14) can be written as:

(16) $$\omega \approx \omega_{0}+\frac{{\left(\gamma D\right)}^{2}}{2\omega_{0}}.$$

This is just the case of a cross-axis sensitivity effect when there is a static magnetic field in the xy-plane.

We can also obtain an approximate solution for $M_{⊥}$ from Equation (13) for only one CARE component, $B_{1}e^{-i\omega_{1}t}$:

(17) $$M_{⊥}=M_{⊥0}+M_{⊥}^{*}\approx \frac{1/T_{2}}{1/T_{2}+\frac{1}{2}\omega_{1}^{2}\frac{1/T_{1}}{{\left(1/T_{1}\right)}^{2}+{\left(\omega -\omega_{n}\right)}^{2}}}M_{⊥0}.$$

where $\omega_{1}=\gamma B_{1}$.

When $\omega_{n}\to \omega_{0}$, Equation (17) becomes:

(18) $$M_{⊥}=\frac{1}{1+\frac{1}{2}\omega_{1}^{2}T_{1}T_{2}}M_{⊥0}.$$

This means that if the CARE magnetic field has the same frequency as $\omega_{0}$, the amplitude of the spin oscillator decreases. However, when $\left|\omega_{n}-\omega_{0}\right|$ is less than a specific value, the spin oscillator will be locked to the CARE field [19]. As a result, Equation (18) will not be valid. This can be seen in the following derivation. We assume there is only one component with frequency $\omega_{r}$ near $\omega_{0}$, so we neglect other components. The CARE magnetic field can be described as:

(19) $$B_{x}^{e}=B_{r}\cos\left(-\omega_{r}t\right),B_{y}^{e}=B_{r}\sin\left(-\omega_{r}t\right),$$

and the feedback magnetic field can be described as:

(20) $$B_{x}^{d}=D\cos\left(-\omega_{r}t-\psi \right),B_{x}^{d}=D\sin\left(-\omega_{r}t-\psi \right).$$

where ψ is a time varying phase.

We use the following expressions for the solutions of the Bloch equation:

(21) $$\begin{array}{l} M_{+}=\left(M_{⊥0}+M_{⊥}^{*}\right)e^{-i\left(\omega_{r}t+\psi \right)}=M_{⊥}e^{-i\left(\omega_{r}t+\psi \right)}\\ M_{z}=M_{z0}+M_{z}^{*} \end{array}.$$

Inserting Equations (19)–(21) into Equation (4), we have:

(22) $$\begin{array}{l} \frac{dM_{+}}{dt}+i\gamma B_{0}M_{+}+\frac{M_{+}}{T_{2}}=i\gamma M_{z}\left(De^{-i\left(\omega_{r}t+\psi \right)}+B_{r}e^{-i\omega_{r}t}\right)\\ \frac{dM_{z}}{dt}=-\gamma D\left(M_{y0}+M_{y}^{*}\right)+\gamma B_{r}\left[\left(M_{x0}+M_{x}^{*}\right)\sin\psi -\left(M_{y0}+M_{y}^{*}\right)\cos\psi \right]+\frac{M_{0}-M_{z}}{T_{1}} \end{array},$$

Substituting Equation (7) into Equation (22), we have:

(23) $$\frac{dM_{z}^{*}}{dt}+\frac{M_{z}^{*}}{T_{1}}=-\gamma DM_{y}^{*}+\gamma B_{r}\left[\left(M_{x0}+M_{x}^{*}\right)\sin\psi -\left(M_{y0}+M_{y}^{*}\right)\cos\psi \right],$$

and:

(24) $$-i\left(\omega_{r}-\omega_{0}+\frac{d\psi }{dt}\right)\left(M_{+0}+M_{+}^{*}\right)+\frac{1}{T_{2}}M_{+}^{*}=i\gamma M_{z}^{*}\left(D+B_{r}e^{i\psi }\right)+i\gamma M_{z0}B_{r}e^{i\psi }.$$

We make the approximation that $d^{2}\psi /dt^{2}=0$ and obtain $M_{z}^{*}$ from Equation (23):

(25) $$M_{z}^{*}=-\gamma DM_{y}^{*}+\frac{\gamma B_{r}}{2\left(\frac{1}{T_{1}}+i\frac{d\psi }{dt}\right)}\left[-iM_{x}^{*}-\left(M_{y0}+M_{y}^{*}\right)\right]e^{i\psi }+\frac{\gamma B_{r}}{2\left(\frac{1}{T_{1}}-i\frac{d\psi }{dt}\right)}\left[iM_{x}^{*}-\left(M_{y0}+M_{y}^{*}\right)\right]e^{-i\psi },$$

Using the approximation that $M_{⊥}^{*}$ varies slowly with time, we obtain the following equation:

(26) $$\begin{array}{l} -i\left(\omega_{r}-\omega_{0}+\frac{d\psi }{dt}\right)\left(M_{+0}+M_{+}^{*}\right)+\frac{1}{T_{2}}M_{+}^{*}=-i{\left(\gamma D\right)}^{2}M_{y}^{*}+i\gamma B_{r}M_{z0}e^{i\psi }-i\gamma^{2}DB_{r}M_{y}^{*}e^{i\psi }\\ +i\frac{\gamma^{2}DB_{r}}{2}\left(\frac{\left[-iM_{x}^{*}-\left(M_{y0}+M_{y}^{*}\right)\right]}{\left(1/T_{1}+i\frac{d\psi }{dt}\right)}e^{i2\psi }+\frac{\left[iM_{x}^{*}-\left(M_{y0}+M_{y}^{*}\right)\right]}{\left(1/T_{1}-i\frac{d\psi }{dt}\right)}\right)\\ +i\frac{{\left(\gamma B_{r}\right)}^{2}}{2}\left(\frac{\left[-iM_{x}^{*}-\left(M_{y0}+M_{y}^{*}\right)\right]}{\left(1/T_{1}+i\frac{d\psi }{dt}\right)}e^{2i\psi }+\frac{\left[iM_{x}^{*}-\left(M_{y0}+M_{y}^{*}\right)\right]}{\left(1/T_{1}-i\frac{d\psi }{dt}\right)}\right) \end{array}.$$

Neglecting the terms containing $e^{i2\psi }$, Equation (26) can be reduced to:

(27) $$\begin{array}{l} -i\left(\omega_{r}-\omega_{0}+\frac{d\psi }{dt}\right)\left(M_{+0}+M_{+}^{*}\right)+\frac{1}{T_{2}}M_{+}^{*}=-i{\left(\gamma D\right)}^{2}M_{y}^{*}+i\gamma B_{r}M_{z0}e^{i\psi }-i\gamma^{2}DB_{r}M_{y}^{*}e^{i\psi }\\ +i\frac{\gamma^{2}DB_{r}}{2}\left(\frac{\left[iM_{x}^{*}-\left(M_{y0}+M_{y}^{*}\right)\right]}{\left(1/T_{1}-i\frac{d\psi }{dt}\right)}\right)+i\frac{{\left(\gamma B_{r}\right)}^{2}}{2}\left(\frac{\left[iM_{x}^{*}-\left(M_{y0}+M_{y}^{*}\right)\right]}{\left(1/T_{1}-i\frac{d\psi }{dt}\right)}\right) \end{array}.$$

This equation can be expressed as:

(28) $$\frac{d\psi }{dt}=\omega_{0}-\omega_{r}+d+l\cos\left(\psi -\psi_{0}\right).$$

where d, l and $\psi_{0}$ are functions of $M_{+0}$, $M_{+}^{*}$, $B_{r}$, $T_{2}$ and $T_{1}$. Equation (28) indicates that the spin oscillator can be locked to the CARE magnetic field when $\left|\omega_{0}-\omega_{r}+d\right|<\left|l\right|$. This phenomenon is similar to the lock-in in ring laser gyroscopes [20,21]. A comprehensive analysis can be seen in [19]. 

We can obtain the lock-in threshold l from Equation (27):

(29) $$l\approx \gamma B_{r}M_{z0}/M_{+0}.$$

The expression of l is consistent with the lock-in threshold with the rotating CARE case given in [19], since we have made rotating wave approximation in this paper. According to [11], the maximum of $M_{+0}$ is $M_{0}^{}\sqrt{T_{2}}/\left(2\sqrt{T_{1}}\right)$ and the corresponding $M_{z0}$ is $M_{0}/2$. Under this condition, we have:

(30) $$l\approx \gamma B_{r}\sqrt{T_{1}/T_{2}}.$$

## 3. Numerical Simulation and Discussion

**Figure 2:**
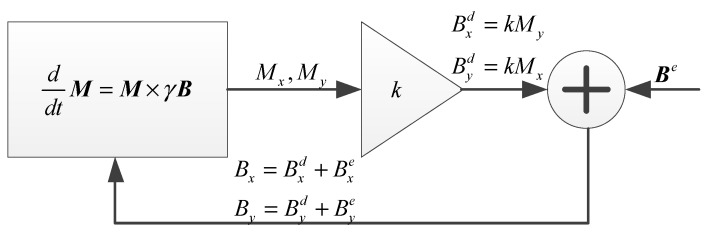
Simulation model corresponding to the NMRG principle given in Figure 1.

We carry out numerical simulation according to Equation (3). The simulation model based on Figure 1 is shown in Figure 2. The feedback magnetic field is $B_{x}^{d}=kM_{y},B_{y}^{d}=kM_{x}$ and the CARE magnetic field is $B_{x}^{e}=B_{r}\cos\left(-\omega_{r}t\right)$, $B_{y}^{e}=B_{r}\sin\left(-\omega_{r}t\right)$. The parameters are as follows: $T_{1}$ = 30 s, $T_{2}$ = 20 s, $B_{0}$ = 1.5 μT, γ=2π × 10 Hz/μT, $M_{z0}$ = 100 A/m, k = 0.0015. The initial values for the magnetization components are $M_{x}=0.01M_{z0}$, $M_{y}=0$ and $M_{z}=M_{z0}$, respectively. Other parameters will be given in the specific simulation. 

The Bloch equations were solved using the MATLAB ODE45 solver. The time step was 50 μs, and the time span was 0–1000 s. When we obtain the magnetization M(t), we use the $M_{y}\left(t\right)$ with a time span of 400 s–1000 s to make a fast Fourier transformation (FFT). We obtain the amplitude and frequency of the spin oscillator according to the peak in the FFT. With a timespan of 600 s, the frequency resolution is 0.0017 Hz and the obtained amplitude also shows errors due to limited data. 

### 3.1. Frequency Shift due to a DC Magnetic Field

**Figure 3:**
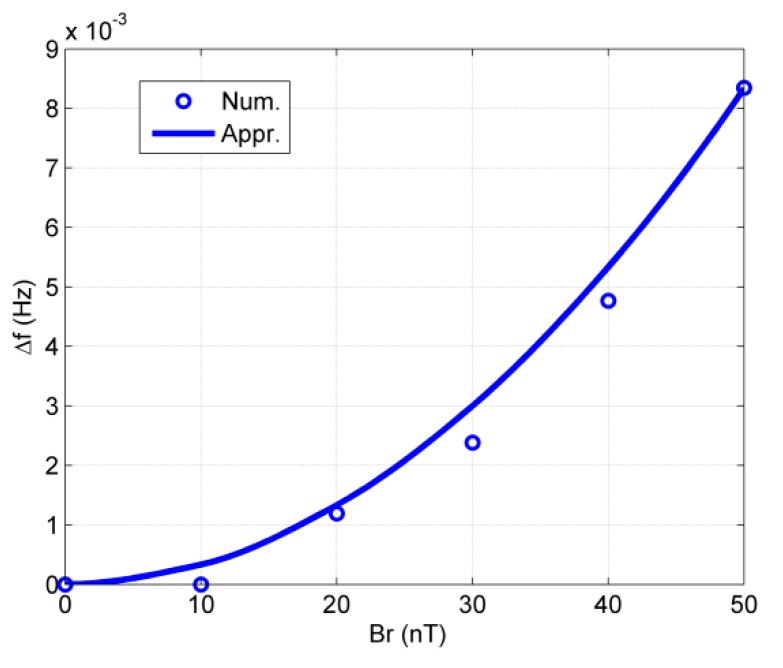
The frequency shift vs. DC magnetic field in the *y*-axis. “Num.” and “Appr.” in the figure denote numerical and approximate analytical results, respectively.

In this case, the CARE magnetic field is $B_{x}^{e}=0,B_{y}^{e}=B_{r}$. In theory, the approximate analytical expression for the oscillation frequency shift will be $\Delta f=\frac{\omega -\omega_{0}}{2\pi }=\frac{1}{2\pi }\frac{{\left(\gamma B_{r}\right)}^{2}}{2\omega_{0}}$. The simulation and approximate results are shown in Figure 3. It is clear that the approximate analytical results agree well with the numerical simulation results.

According to $B_{y}^{eq}=\Omega_{y}/\gamma$ we know that the rotation for the *y*-axis will lead to an equivalent magnetic field. When the NMRG is placed in a vehicle, the maximum value of Ω_*y*_ can be as high as 2*π* rad/s, equivalent to a magnetic field of 78 nT for the ^129^Xe spin oscillator and 284 nT for the ^131^Xe spin oscillator. When $B_{0}$ = 12 μT, the frequency shifts for ^129^Xe and ^131^Xe are 3.5 mHz and 11.8 mHz, respectively. This is a huge error for the NMRG, since it should measure Ω_*z*_ with an error of less than 10 nHz for typical applications. To suppress this cross-axis effect, some methods can be used. For example, with a three-dimensional magnetic field compensation [22], the influence of low frequency Ω_*y*_ can be suppressed greatly, but the bandwidth of the feedback loop should be as high as possible.

### 3.2. Frequency Shift due to an Oscillating Magnetic Field

#### 3.2.1. One Component

**Figure 4:**
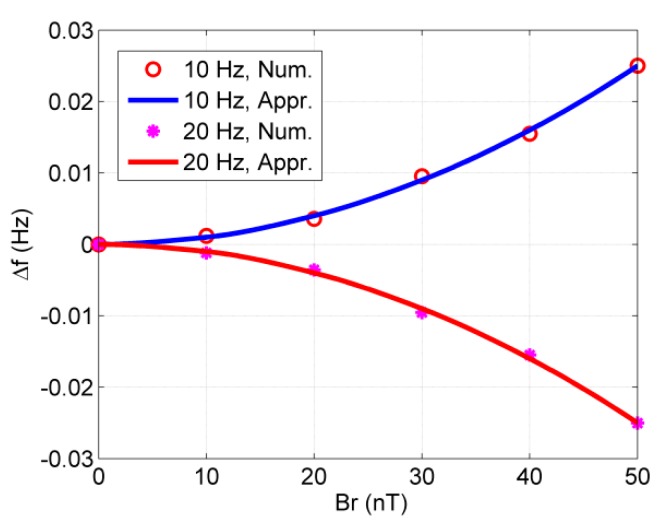
The frequency shift vs. *B_r_* for different frequencies of the Cross-Axis-Rotation-Equivalent (CARE) magnetic field.

At first, we carry out simulations for the CARE magnetic field with only one frequency, where $B_{x}^{e}=B_{r}\cos\left(-\omega_{r}t\right),B_{y}^{e}=B_{r}\sin\left(-\omega_{r}t\right)$. According to a previous analysis, the approximate frequency shift will be $\Delta f=\frac{1}{4\pi }\gamma^{2}B_{r}^{2}\frac{1}{\omega_{0}-\omega_{r}}$. We choose two $\omega_{r}$ values and make the Δf curve a function of $B_{r}$. The simulation and approximate results are shown in Figure 4. The approximate analytical results agree well with the numerical simulation results.

**Figure 5:**
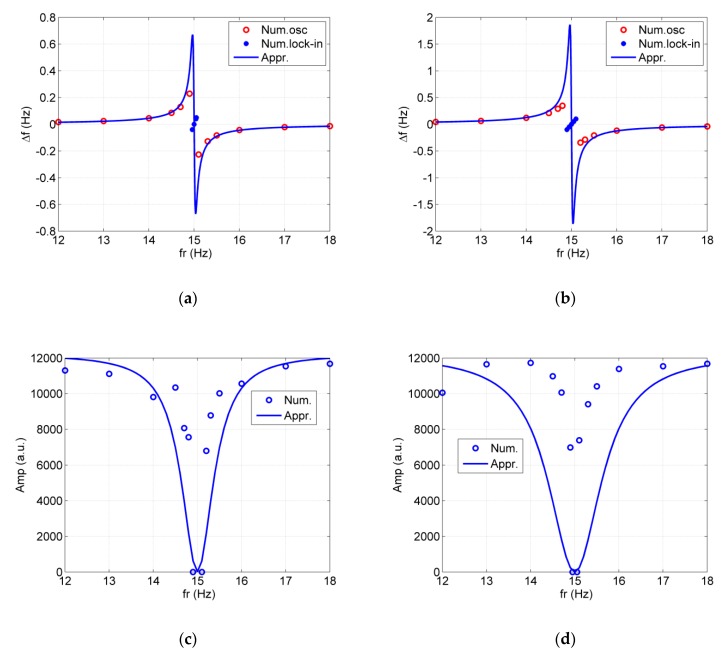
The frequency shift Δf and amplitude Amp of the spin oscillator vs. frequencies of the CARE magnetic field. (**a**) Δf vs. $f_{r}$ for $B_{r}$ = 30 nT; (**b**) Δf vs. $f_{r}$ for $B_{r}$ = 50 nT; (**c**) Amp vs. $f_{r}$ for $B_{r}$ = 30 nT; (**d**) Amp vs. $f_{r}$ for $B_{r}$ = 50 nT. In (**a**,**b**), Num. lock-in denotes the frequency shift in the lock-in state. In the lock-in state of (**c**,**d**), we let Amp = 0 to express that the spin oscillator cannot normally measure the rotation rates. In fact, the amplitude and frequency of the spin oscillator in the lock-in state is governed by the CARE magnetic field. The limited data for FFT contribute to the scattering of frequency shift and Amp to some extent.

Next, at a specific magnetic field $B_{r}$, we change the frequency $f_{r}$. The frequency shift $\Delta f=\frac{1}{4\pi }\frac{\gamma^{2}B_{r}^{2}}{\omega_{0}-\omega_{r}}$ and amplitude $Amp=\frac{1/T_{2}}{1/T_{2}+\frac{1}{2}\gamma^{2}B_{r}^{2}\frac{1/T_{1}}{{\left(1/T_{1}\right)}^{2}+{\left(\omega -\omega_{n}\right)}^{2}}}Amp_{0}$ of the spin oscillator are given in Figure 5. Here, $Amp_{0}$ denotes the amplitude of the spin oscillator without a CARE field. When $\omega_{r}\to \omega_{0}$, |Δf| becomes very large. The relaxation time $T_{1}$ removes the frequency shift singular point when $\omega_{r}\to \omega_{0}$. At a specific $B_{r}$, the spin oscillator is locked to the CARE magnetic field and its frequency is equal to $\omega_{r}$. We also find that when $\omega_{r}$ approaches $\omega_{0}$, the approximation solution has a larger error. The reason for this is that when $\omega_{r}$ approaches $\omega_{0}$, we omitted some terms containing $e^{i\left(\omega -\omega_{n}\right)t}$ in the derivation for Equations (11) and (13). The approximation for Amp has a slightly larger error, but it still agrees with the numerical simulation qualitatively. That is, the CARE magnetic field near the Larmor frequency reduces the amplitude of the spin oscillator. It should be noted that the simulation program from Equation (3) also contributes to some error, which leads to the numerical data dispersion shown in Figure 5c,d.

#### 3.2.2. Two Harmonic Components

**Figure 6:**
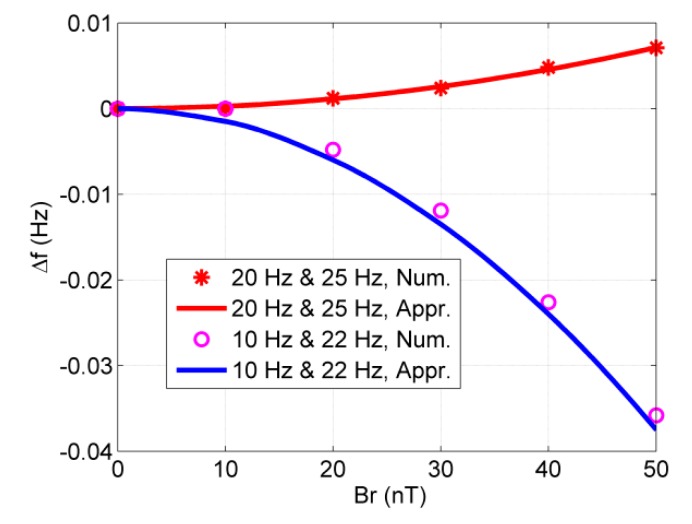
The frequency shift vs. magnetic field amplitude for different groups of magnetic fields. Two groups of magnetic field are given: one is $f_{r1}=20$ Hz and $f_{r2}=25$ Hz while another is 10 Hz and $f_{r2}=22$ Hz.

To check the approximate frequency shift in Equation (14) when the CARE magnetic field has more frequency components, we choose $B_{x}^{e}=B_{r1}\cos\left(-\omega_{r1}t\right)+B_{r2}\cos\left(-\omega_{r2}t\right)$ and $B_{y}^{e}=B_{r1}\sin\left(-\omega_{r1}t\right)+B_{r2}\sin\left(-\omega_{r2}t\right)$ to carry out numerical simulations. The approximate frequency shift is $\Delta f=\frac{1}{4\pi }\gamma_{}^{2}B_{r}^{2}\left(\frac{1}{\omega_{0}-\omega_{r1}}+\frac{1}{\omega_{0}-\omega_{r2}}\right)$. Here, we choose $B_{r1}=B_{r2}=B_{r}$. The results are shown in Figure 6, which shows that the approximate frequency shift agrees well with the numerical simulation.

### 3.3. Discussions

#### 3.3.1. Comparing the ^3^He Gyroscope with a Dual-isotope Xe NMRG

Both the amplitude and frequency of the spin oscillator are changed due to the CARE magnetic field. The dynamical equation for analyzing the ^3^He gyroscope omitted the relaxation times T_1_ and T_2_ in Equation (3) and did not need the driving magnetic field. Therefore, the two types of gyroscopes are expected to show different behavior.

For a CARE magnetic field with very low frequency, the frequency shift for the two gyroscopes can be described by Equation (16). However, when the frequency of the CARE magnetic field is near the Larmor frequency, the ^3^He gyroscope loses signal periodically, and the amplitude of the Xe NMRG signal decreases. For the oscillation frequency of the Xe spin oscillator, it is locked to the frequency of the CARE magnetic field, and, out of the lock-in range, the oscillation frequency shifts according to Equation (14). This shows the CARE magnetic field pushes the oscillation frequency of a spin oscillator far away from the CARE magnetic field frequency. Therefore, the CARE magnetic field cannot be common-mode suppressed effectively by a dual-isotope scheme.

#### 3.3.2. Methods to Reduce the Cross-Axis Effect

If the CARE magnetic field results from the rotation of a vehicle, it has a low frequency. Therefore, it is clear that we can reduce the cross-axis effect by increasing $B_{0}$. Assuming a cross-axis input rotation rate of 1 rad/s, $B_{0}$ should be larger than 0.049 T for a ^3^He gyroscope [10]. This is not practical, since the magnetic inhomogeneity can be very large at this magnetic field. Fortunately, the transverse magnetic field in Xe NMRGs can be compensated by an alkali atom magnetometer and a feedback loop [22]. In general, a compensation bandwidth of approximately 10 Hz can be obtained.

For a high-frequency CARE magnetic field, the compensation loop cannot follow it in a timely manner. If the frequency spectrum of the angular vibration covers the Larmor frequency of the spin oscillator, it will cause a severe error. We might as well take the rotation component around the *y*-axis as an example to discuss the influence of the angular vibration [23,24]. We assume the rotation component has an amplitude of 0.003 rad/s at 100 Hz, corresponding to $B_{y}^{eq}=0.04$ nT for ^129^Xe and $B_{y}^{eq}=0.13$ nT for ^131^Xe. When $B_{0}=10$ μT, the frequency shifts of ^129^Xe and ^131^Xe due to the CARE magnetic field are 12.8 μHz and −3.7 μHz, respectively. Moreover, the worst case is a lock-in phenomenon, which makes the NMRG unresponsive toward $\Omega_{z}$. With $T_{1}=T_{2}=25$ s, $\gamma B_{r}=0.003$ rad/s and the lock-in threshold is approximately 0.17 °/s. It is a severe error for an NMRG.

To improve the tolerance of the NMRG toward the cross-axis angular vibration, it is better to choose a stronger $B_{0}$, to ensure that the Larmor frequency is far away from the angular vibration component. The system level vibration isolation can also be used to attenuate possible angular vibration. In order to solve the lock-in problem, we can also use the biasing method just as that in ring laser gyroscopes, but it will make the NMRG more complicated. 

## 4. Conclusions

In summary, we analyzed the response of a spin oscillator to a magnetic field in the xy-plane with both analytical and numerical methods. Approximate analytical equations for the frequency shift and amplitude of the spin oscillator with the CARE field are obtained, which are verified by solving the Bloch equations numerically. Then, we discussed the measurement error in an NMRG due to a rotation component in the x or *y*-axis. The NMRG is a single-axis gyroscope, but it also has cross-axis sensitivity to the x and *y*-axis. If the NMRG is subject to cross-axis rotation with a frequency near to its magnetic resonance frequency, the measurement error is relatively large, and the rotation rate cannot be measured. To reduce the cross-axis coupling effect, the resonance magnetic field should be as large as possible and a mechanical notch filter covering the oscillating frequencies of ^129^Xe and ^131^Xe can be used. These problems should be considered in the design of NMRG-based strap-down inertial navigation systems.
